# Finite-Time *H*_∞_ Controllers Design for Stochastic Time-Delay Markovian Jump Systems with Partly Unknown Transition Probabilities

**DOI:** 10.3390/e26040292

**Published:** 2024-03-27

**Authors:** Xinye Guo, Yan Li, Xikui Liu

**Affiliations:** 1 College of Mathematics and Systems Science, Shandong University of Science and Technology, Qingdao 266590, China; guoxyqd@163.com; 2 Department of Fundamental Courses, Shandong University of Science and Technology, Jinan 250031, China; 3 Department of Electrical Engineering and Information Technology, Shandong University of Science and Technology, Jinan 250031, China

**Keywords:** Markovian jump systems, discrete-time systems, finite-time control, *H*_∞_ control, partly unknown transition probabilities

## Abstract

This paper concentrates on the finite-time $H_{\infty }$ control problem for a type of stochastic discrete-time Markovian jump systems, characterized by time-delay and partly unknown transition probabilities. Initially, a stochastic finite-time (SFT) $H_{\infty }$ state feedback controller and an SFT $H_{\infty }$ observer-based state feedback controller are constructed to realize the closed-loop control of systems. Then, based on the Lyapunov–Krasovskii functional (LKF) method, some sufficient conditions are established to guarantee that closed-loop systems (CLSs) satisfy SFT boundedness and SFT $H_{\infty }$ boundedness. Furthermore, the controller gains are obtained with the use of the linear matrix inequality (LMI) approach. In the end, numerical examples reveal the reasonableness and effectiveness of the proposed designing schemes.

## 1. Introduction

The structure or parameters of various practical systems often undergoes changes due to environmental mutations, component failures, and other factors, resulting in a decrease in system performance and potential instability [1]. How to ensure the stability of mutation systems has been one of the hot topics for scholars. Markovian jump systems (MJSs), a type of hybrid systems consisting of several subsystems, can be used to model dynamical systems with structural mutations and have been extensively researched in both the practical and theoretical domains [2,3]. An adaptive neural network-based control approach was devised in [4] to address the problem of fault-tolerant control for nonlinear MJSs. In [5], the asynchronous filtering problem of MJSs affected by time-varying and infinite distributed delays was studied by using the homogeneous polynomial method. For stochastic T-S fuzzy singular MJSs, the robust $H_{\infty }$ sliding mode control problem was studied in [6,7]. The authors of [8] studied the fault-detection filter design problem of uncertain singular MJSs by means of the LKF and convex polyhedron techniques. In addition, for the achievements regarding the stability and stabilization of MJSs, readers may see [9,10] and references therein.

It should be emphasized that there is a qualification in references [4,5,6,7,8,9,10], that is, the transition probability (TP) information of MJSs must be exactly and completely known. However, due to the limitations of measurement costs and measuring instruments, this condition is difficult to meet in the actual system modeling. As a result, it is essential and significant to investigate MJSs with partly unknown TPs [11]. For networked MJSs with partly unknown TPs, the event-triggered dynamic output feedback control problem and sliding mode control problem were solved in [12,13], respectively. For a type of singular MJSs with partly unknown TPs, the $H_{\infty }$ filtering problem was studied in [14,15]. The authors of [16] achieved the event-triggered guaranteed cost control for time-delay MJSs with partly unknown TPs, and some sufficient conditions were established to guarantee the presence of guaranteed cost controllers. In [17], a state feedback controller was constructed to ensure that MJSs with partly unknown TPs were stochastic stable. A sliding mode controller based on an adaptive neural network was proposed in [18], and the reliable control problem of uncertain MJSs with partially unknown TPs was studied.

Notably, most of the above research findings mainly concentrate on the asymptotic behavior of systems in an infinite-time interval, namely, as in the Lyapunov stability theory. However, in many practical systems, such as vehicle emergency braking systems [19], aircraft-tracking systems [20], and ship-maneuvering systems [21], it is required that the systems respond ideally to work in a finite time interval. To realize this practical need, in 1961, Dorato proposed finite-time stability [22]. Since finite-time stability has a better transient performance, a faster response speed, and a higher tracking accuracy, it has been applied to MJSs [23,24,25,26], T-S fuzzy systems [27,28,29], nonlinear pulse systems [30,31], mean-field systems [32,33,34], and so on.

In addition to requiring better transient performance, modern industries increasingly emphasize the anti-interference performance of control systems. Both external disturbances and imprecise modeling can adversely affect the performance of control systems. To weaken the effect of external disturbances, $H_{\infty }$ control has emerged. Recently, many scholars have carried out plenty of research on finite-time $H_{\infty }$ control [35,36,37,38,39,40,41]. Specifically, ref. [35] introduced a new switching dynamic event-triggering mechanism, and discussed the finite-time $H_{\infty }$ control problem for switching fuzzy systems. In [36], the finite-time $H_{\infty }$ control problem of nonlinear pulse switching systems was studied to guarantee that the CLS was bounded. On the other hand, due to the constraints of measurement technology and measurement costs, the system state information is frequently challenging to measure directly. In tackling this challenge, many meaningful results of finite-time $H_{\infty }$ observer-based controller designing schemes have been successfully attained; see [37,38,39,40,41] and references therein.

At present, the study of continuous-time MJSs has obtained rich results. With the popularization of digital controllers and the development of computer science and technology, the research on discrete-time systems has attracted much attention. Discrete-time MJSs provide a framework for modeling and analyzing a variety of complex systems in the real world [42]. Through the discrete description of the system, it is easier to analyze the dynamic behavior, stability, and convergence of the system [43]. This kind of modeling and analysis is essential for understanding and predicting the behavior of systems [44], and is widely used in control systems.

Inspired by the preceding analysis, this article presents the designing schemes of a stochastic finite-time $H_{\infty }$ state feedback controller and a stochastic finite-time $H_{\infty }$ observer-based state feedback controller for a discrete-time MJS. Different from [17,38], the MJS considered in this paper is influenced by a time delay and stochastic white noise, which is more in line with the actual demand, but also increases the difficulty of the article derivation. Compared with the existing literature, the primary contributions of this study include the following:

(I) In this paper, the state feedback control strategy and the observer-based state feedback control strategy are adopted. The concepts of SFT $H_{\infty }$ state feedback stabilization and SFT $H_{\infty }$ observer-based state feedback stabilization for time-delay MJSs are defined simultaneously for the first time. The results of [17,38,44] are extended to time-delay MJSs with partially unknown TPs.

(II) By constructing a delay-dependent LKF, several sufficient conditions are given to ensure that the CLS is SFT $H_{\infty }$-bounded under two control strategies.

The article is structured as follows: Section 2 presents an introduction to the system along with some preliminary knowledge. In Section 3, a state feedback controller is designed, and some sufficient conditions for the MJS to be SFT $H_{\infty }$ state feedback stabilization are obtained through the LKF and LMI methods. Similar to Section 3, in Section 4 we design an observer-based state feedback controller and verify that the MJS is SFT $H_{\infty }$ observer-based state feedback stabilization. In Section 5, the feasibility and effectiveness of this work are validated through two simulation examples. Section 6 summarizes the entire article and provides an outlook on future research directions.

Notation: $A^{-1}$ and $A^{T}$ represent the matrix inverse and transpose of matrix *A*, respectively. The expression for a real positive definite matrix *A* is denoted as A>0. $diag\{P_{1},P_{2},\cdots ,P_{n}\}$ is the block diagonal matrix with $P_{1},P_{2},\cdots ,P_{n}$ on the diagonal. We denote $I_{n\times n}$ as the identity matrix with n×n dimensions. $N^{+}$ is the set of positive integers and R is the real number set. $R^{m}$ and $R^{m\times n}$ are the *m*-dimensional Euclidean space with 2-norm ∥·∥ and the vector space of all m×n matrices with entries in R, respectively. E{σ} represents the mathematical expectation of σ. The symbol ∗ implies the symmetric hidden matrix entries. This paper presupposes that every specified matrix possesses the necessary dimensions. For ease of understanding, the acronyms in this paper and their corresponding meanings are shown in Table 1.

## 2. System Description and Preliminary Knowledge

Consider an MJS with a time delay, as outlined below:

(1) $$\left\{\begin{array}{cc} x(k+1)\;=\; & A_{1}\left(m_{k}\right)x\left(k\right)+A_{d1}\left(m_{k}\right)x\left(k-\tau \right)+B_{1}\left(m_{k}\right)u\left(k\right)+C_{1}\left(m_{k}\right)v\left(k\right)\\ \; & +\left[A_{2}\left(m_{k}\right)x\left(k\right)+A_{d2}\left(m_{k}\right)x\left(k-\tau \right)+B_{2}\left(m_{k}\right)u\left(k\right)+C_{2}\left(m_{k}\right)v\left(k\right)\right]\omega \left(k\right),\\ y(k)\;=\; & D\left(m_{k}\right)x\left(k\right)+G\left(m_{k}\right)u\left(k\right),\\ z(k)\;=\; & D_{1}\left(m_{k}\right)x\left(k\right)+D_{d1}\left(m_{k}\right)x\left(k-\tau \right)+G_{1}\left(m_{k}\right)u\left(k\right)+G_{2}\left(m_{k}\right)v\left(k\right),\;\;k\in \left\{0,1,2,\cdots ,\hat{T}\right\},\\ x(n)\;=\; & \psi (n),\;\;n\in \{-\tau ,-\tau +1,\cdots ,0\}, \end{array}\right.$$

where $x\left(k\right)\in R^{n}$ is the system state, $y\left(k\right)\in R^{p}$ is the measured output, $z\left(k\right)\in R^{r}$ is the control output, and $u\left(k\right)\in R^{q}$ is the control input. ψ(n),n∈{−τ,−τ+1,⋯,0} are the initial conditions. τ is a positive integer that signifies the fixed time delay. The sequence ω(k) denotes one-dimensional white noises on the complete probability space (Ω,F,P), and satisfies E{ω(k)}=0 and $E\left\{\omega \left(k\right)\omega \left(s\right)\right\}=\delta_{ks}$, where $\delta_{ks}$ is the Kronecker delta. $v\left(k\right)\in R^{l}$ stands for the external disturbance, which satisfies the following:

(2) $$\sum_{k=0}^{\hat{T}}v^{T}\left(k\right)v\left(k\right)\leq h,\;h\geq 0.$$

$A_{1}\left(m_{k}\right)$, $A_{2}\left(m_{k}\right)$, $A_{d1}\left(m_{k}\right)$, $A_{d2}\left(m_{k}\right)$, $B_{1}\left(m_{k}\right)$, $B_{2}\left(m_{k}\right)$, $C_{1}\left(m_{k}\right)$, $C_{2}\left(m_{k}\right)$, $D(m_{k})$, $D_{1}\left(m_{k}\right)$, $D_{d1}\left(m_{k}\right)$, $G(m_{k})$, $G_{1}\left(m_{k}\right)$, and $G_{2}\left(m_{k}\right)$ are coefficient matrices with appropriate dimensions. These matrices depend upon the Markovian jump process $\left\{m_{k},k\geq 0\right\}$, which is a discrete-time, discrete-state Markovian chain taking values in a finite state space S={1,2,⋯,N} with transition probabilities $\pi_{ij}$, where $\pi_{ij}=Pr\left\{m_{k+1}=j|m_{k}=i\right\},\;i,j\in S$, denotes the transition probability from mode *i* at time *k* to mode *j* at time k+1, and satisfies $\sum_{j=1}^{N}\pi_{ij}=1,\pi_{ij}\geq 0\;\left(i\in S\right)$. When $m_{k}=i,\;i\in S$, the system parameter matrices are expressed by $A_{1i}$, $A_{2i}$, $A_{d1i}$, $A_{d2i}$, $B_{1i}$, $B_{2i}$, and so on. In addition, ω(k) and $m_{k}$ are independent of each other.

In this paper, it is presumed that the information in the TP matrix is partially available. In this situation, the TP matrix Π for an MJS with *N* modes may be represented by:

(3) $$\Pi =\left[\begin{array}{cccc} {\hat{\pi }}_{11} & \pi_{12} & \cdots & \pi_{1N}\\ {\hat{\pi }}_{21} & \pi_{22} & \cdots & {\hat{\pi }}_{2N}\\ \vdots & \cdots & \ddots & \vdots \\ {\hat{\pi }}_{N1} & \pi_{N2} & \cdots & \pi_{NN} \end{array}\right],$$

where ${\hat{\pi }}_{ij}$ is the unknown TP, for all i∈S, and the set *S* is defined as $S=S_{k}^{i}\cup S_{uk}^{i}$, where:

(4) $$S_{k}^{i}=\left\{j:\pi_{ij}\;is\;known\right\},\;S_{uk}^{i}=\left\{j:\pi_{ij}\;is\;unknown\right\}.$$

Moreover, when $S_{k}^{i}\neq \emptyset$, then it can be described as:

(5) $$S_{k}^{i}=\left\{\zeta_{1},\zeta_{2},\cdots ,\zeta_{p_{i}}\right\},\;p_{i}\in \left\{1,2,\cdots ,N-2\right\},$$

where $\zeta_{g}\in N^{+},\;g\in \left\{1,2,\cdots ,p_{i}\right\}$ denotes the *g*-th known element in the *i*-th row of the TP matrix Π. Similarly, when $S_{uk}^{i}\neq \emptyset$, it can be expressed as follows:

(6) $$S_{uk}^{i}=\left\{\zeta_{u1},\zeta_{u2},\cdots ,\zeta_{uq_{i}}\right\},\;q_{i}\in \left\{2,\cdots ,N\right\},$$

where $\zeta_{ug}\in N^{+},\;g\in \left\{1,2,\cdots ,q_{i}\right\}$ is the *g*-th unknown element in the *i*-th row of the TP matrix Π.

**Remark** **1.** *Since $\sum_{j=1}^{N}\pi_{ij}=1$, there are at least two unknown elements in (3), and if there are unknown elements in a certain row, their quantity is at least two.*

**Lemma** **1.** *(Schur’s complement [38]) The LMI*

$$S=\left[\begin{array}{cc} S_{11} & S_{12}^{T}\\ S_{12} & S_{22} \end{array}\right]<0$$

*is equivalent to $S_{11}-S_{12}^{T}S_{22}^{-1}S_{12}<0$, where $S_{22}<0$.*

**Definition** **1.** *(SFT stability)*

*The MJS (1) with v(k)=0 is said to be SFT-stable with respect to $\left(\rho_{1},\rho_{2},R_{i},\hat{T}\right)$, if:*

(7) $$\underset{k_{0}\in \left\{-\tau ,\cdots ,0\right\}}{\sup}E\left\{x^{T}\left(k_{0}\right)R_{i}x\left(k_{0}\right)\right\}\leq \rho_{1}\Rightarrow E\left\{x^{T}\left(k\right)R_{i}x\left(k\right)\right\}<\rho_{2},\;\;\forall k\in \left\{0,1,2,\cdots ,\hat{T}\right\}$$

*holds for matrix $R_{i}>0,\;i\in S$, and given scalars $0<\rho_{1}<\rho_{2}$.*

**Remark** **2.** *Definition 1 means that if the initial state is bounded, then the state trajectory of the system does not exceed a predetermined boundary in a finite time interval under certain conditions, which is different from asymptotic stability. An asymptotically stable system may not be finite-time stable, if its state trajectory exceeds the given upper bound in a finite-time interval, and vice versa.*

**Definition** **2.** *(SFT boundedness)*

*The MJS (1) is said to be SFT-bounded with respect to $(\rho_{1},\rho_{2},$ $R_{i},\hat{T},h)$ if the system state x(k) and the external disturbance v(k) satisfy (7) and (2), respectively.*

## 3. Finite-Time $H_{\infty }$ State Feedback Control

### 3.1. State Feedback Controller

We design the following state feedback controller for MJS (1):

(8) $$u\left(k\right)=K\left(m_{k}\right)x\left(k\right),$$

where $K(m_{k})$ is the state feedback controller gain to be designed and $K(m_{k})$ is denoted by $K_{i}$ when $m_{k}=i,i\in S$. Then, the resulting CLS can be described as follows:

(9) $$\left\{\begin{array}{cc} x(k+1)\;=\; & {\bar{A}}_{1i}x\left(k\right)+A_{d1i}x\left(k-\tau \right)+C_{1i}v\left(k\right)\\ \; & +[{\bar{A}}_{2i}x\left(k\right)+A_{d2i}x\left(k-\tau \right)+C_{2i}v\left(k\right)]\omega \left(k\right),\\ z(k)\;=\; & {\bar{D}}_{1i}x\left(k\right)+D_{d1i}x\left(k-\tau \right)+G_{2i}v\left(k\right), \end{array}\right.$$

where ${\bar{A}}_{1i}=A_{1i}+B_{1i}K_{i}$,  ${\bar{A}}_{2i}=A_{2i}+B_{2i}K_{i}$,  ${\bar{D}}_{1i}=D_{1i}+G_{1i}K_{i}$.

**Definition** **3.** *(SFT $H_{\infty }$ boundedness)*

*The CLS (9) is said to be SFT $H_{\infty }$-bounded with respect to $\left(\rho_{1},\rho_{2},R_{i},\hat{T},h,\gamma \right)$ if the subsequent two conditions hold:*

*(a)*  *The CLS (9) satisfies SFT boundedness with respect to $\left(\rho_{1},\rho_{2},R_{i},\hat{T},h\right)$;*
*(b)*  *Under the zero initial condition, for any external disturbance v(k) satisfying (2), the control output z(k) satisfies*

(10) $$E\left\{\sum_{k=0}^{\hat{T}}z^{T}\left(k\right)z\left(k\right)\right\}<\gamma^{2}E\left\{\sum_{k=0}^{\hat{T}}v^{T}\left(k\right)v\left(k\right)\right\},$$

*where $R_{i}>0,\;i\in S$, γ>0, $0<\rho_{1}<\rho_{2}$.*

**Definition** **4.** *(SFT $H_{\infty }$ state feedback stabilization)*

*The MJS (1) is said to be SFT $H_{\infty }$ state feedback stabilization with respect to $(\rho_{1},\rho_{2},R_{i},\hat{T},$ h,γ) if there exists a state feedback controller (8) such that the CLS (9) satisfies SFT $H_{\infty }$ boundedness. Moreover, the controller (8) is called the SFT $H_{\infty }$ state feedback controller.*

### 3.2. Main Results

This section will present some sufficient conditions for the existence of a state feedback controller (8) for system (1).

**Theorem** **1.** *The CLS (9) with partly unknown TPs is SFT $H_{\infty }$-bounded with respect to $(\rho_{1},\rho_{2},R_{i},$ $\hat{T},h,\gamma )$ if there exist the scalars α>1 and γ>0 and matrices M>0 and $P_{i}>0$ for all i∈S, satisfying the following:*

(11) $$\left[\begin{array}{ccc} \Theta_{1i}^{T}-\alpha P_{i}+M+{\bar{D}}_{1i}^{T}{\bar{D}}_{1i} & \Theta_{2i}^{T}+{\bar{D}}_{1i}^{T}D_{d1i} & \Theta_{3i}^{T}+{\bar{D}}_{1i}^{T}G_{2i}\;\\ {}* & \Theta_{4i}^{T}-M+D_{d1i}^{T}D_{d1i} & \Theta_{5i}^{T}+D_{d1i}^{T}G_{2i}\;\\ {}* & * & \Theta_{6i}^{T}-\gamma^{2}I+G_{2i}^{T}G_{2i} \end{array}\right]<0,$$

(12) $$\alpha^{\hat{T}}\rho_{1}\left[\underset{i\in S}{\sup}\left\{\lambda_{max}\left({\bar{P}}_{i}\right)\right\}+\underset{i\in S}{\sup}\left\{\lambda_{max}\left({\bar{M}}_{i}\right)\right\}\tau \right]+\gamma^{2}\alpha^{\hat{T}}h<\underset{i\in S}{\inf}\left\{\lambda_{min}\left({\bar{P}}_{i}\right)\right\}\rho_{2},$$

*where $\Theta_{1i}^{T}={\bar{A}}_{1i}^{T}\Psi_{i}{\bar{A}}_{1i}+{\bar{A}}_{2i}^{T}\Psi_{i}{\bar{A}}_{2i}$,  $\Theta_{2i}^{T}={\bar{A}}_{1i}^{T}\Psi_{i}A_{d1i}+{\bar{A}}_{2i}^{T}\Psi_{i}A_{d2i}$,*

*$\Theta_{3i}^{T}={\bar{A}}_{1i}^{T}\Psi_{i}C_{1i}+{\bar{A}}_{2i}^{T}\Psi_{i}C_{2i}$,  $\Theta_{4i}^{T}=A_{d1i}^{T}\Psi_{i}A_{d1i}+A_{d2i}^{T}\Psi_{i}A_{d2i}$,*

*$\Theta_{5i}^{T}=A_{d1i}^{T}\Psi_{i}C_{1i}+A_{d2i}^{T}\Psi_{i}C_{2i}$,  $\Theta_{6i}^{T}=C_{1i}^{T}\Psi_{i}C_{1i}+C_{2i}^{T}\Psi_{i}C_{2i}$,*

*$\Psi_{i}=\sum_{j\in S_{k}^{i}}\pi_{ij}P_{j}+\left(1-\pi_{k}^{i}\right)\left(\sum_{j\in S_{uk}^{i}}P_{j}\right)$,  ${\bar{P}}_{i}=R_{i}^{-\frac{1}{2}}P_{i}R_{i}^{-\frac{1}{2}},\;{\bar{M}}_{i}=R_{i}^{-\frac{1}{2}}MR_{i}^{-\frac{1}{2}}$.*

**Proof.**  For the CLS (9), we consider the following LKF:

(13) $$V\left(x\left(k\right),m_{k}=i\right)=x^{T}\left(k\right)P_{i}x\left(k\right)+\sum_{l=k-\tau }^{k-1}x^{T}\left(l\right)Mx\left(l\right).$$

Then, we compute the following:

(14) $$\begin{array}{cc} E\{\Delta V(x\left(k\right),m_{k}=i)\}= & E\left\{V\left(x\left(k+1\right),m_{k+1}=j\right)\right\}-E\left\{V\left(x\left(k\right),m_{k}=i\right)\right\}\\ = & \sum_{j\in S}\pi_{ij}x^{T}\left(k+1\right)P_{j}x\left(k+1\right)+\sum_{l=k+1-\tau }^{k}x^{T}\left(l\right)Mx\left(l\right)\\ \; & -x^{T}\left(k\right)P_{i}x\left(k\right)-\sum_{l=k-\tau }^{k-1}x^{T}\left(l\right)Mx\left(l\right)\\ = & x^{T}\left(k+1\right)\sum_{j\in S}\pi_{ij}P_{j}x\left(k+1\right)+x^{T}\left(k\right)\left[M-P_{i}\right]x\left(k\right)-x^{T}\left(k-\tau \right)Mx\left(k-\tau \right). \end{array}$$

Since the TP matrix Π contains partly accessible information, not all the probabilities $\pi_{ij}\;\left(j\in S\right)$ are known. Thus, we denote $\pi_{k}^{i}=\sum_{j\in S_{k}^{i}}\pi_{ij}$. ${\hat{\pi }}_{ij}$ are the unknown TPs of Π. Moreover, from $\sum_{j=1}^{N}\pi_{ij}=1$, it is obvious that $\sum_{j\in S_{uk}^{i}}{\hat{\pi }}_{ij}=1-\pi_{k}^{i}\geq 0$. Supposing that $\pi_{k}^{i}<1$, we can obtain the following:

(15) $$\begin{array}{cc} \sum_{j\in S}\pi_{ij}P_{j}= & \sum_{j\in S_{k}^{i}}\pi_{ij}P_{j}+\sum_{j\in S_{uk}^{i}}{\hat{\pi }}_{ij}P_{j}\\ = & \sum_{j\in S_{k}^{i}}\pi_{ij}P_{j}+\left(1-\pi_{k}^{i}\right)\sum_{j\in S_{uk}^{i}}\frac{{\hat{\pi }}_{ij}}{\left(1-\pi_{k}^{i}\right)}P_{j}\\ \leq & \sum_{j\in S_{k}^{i}}\pi_{ij}P_{j}+\left(1-\pi_{k}^{i}\right)\left(\sum_{j\in S_{uk}^{i}}P_{j}\right)=\Psi_{i}. \end{array}$$

By (15), we can rewrite (14) as follows:

(16) $$\begin{array}{c} E\left\{\Delta V(x\left(k\right),m_{k}=i)\right\}\leq x^{T}\left(k+1\right)\Psi_{i}x\left(k+1\right)+x^{T}\left(k\right)\left[M-P_{i}\right]x\left(k\right)-x^{T}\left(k-\tau \right)Mx\left(k-\tau \right)\\ ={\left[\begin{array}{c} x(k)\\ x(k-\tau )\\ v(k) \end{array}\right]}^{T}\left[\begin{array}{ccc} \Theta_{1i}^{T}-P_{i}+M & \Theta_{2i}^{T} & \Theta_{3i}^{T}\;\\ {}* & \Theta_{4i}^{T}-M & \Theta_{5i}^{T}\;\\ {}* & * & \Theta_{6i}^{T} \end{array}\right]\left[\begin{array}{c} x(k)\\ x(k-\tau )\\ v(k) \end{array}\right].\; \end{array}$$

From (16) and (11), we have the following:

$$\begin{array}{cc} E\{\Delta V(x\left(k\right),m_{k}=i)\}< & \left(\alpha -1\right)E\left\{x^{T}\left(k\right)P_{i}x\left(k\right)\right\}+\gamma^{2}E\left\{v^{T}\left(k\right)v\left(k\right)\right\}-E\left\{z^{T}\left(k\right)z\left(k\right)\right\}\\ < & \left(\alpha -1\right)E\left\{x^{T}\left(k\right)P_{i}x\left(k\right)\right\}+\gamma^{2}E\left\{v^{T}\left(k\right)v\left(k\right)\right\}\\ \leq & \left(\alpha -1\right)E\left\{V\left(x\left(k\right),m_{k}=i\right)\right\}+\gamma^{2}E\left\{v^{T}\left(k\right)v\left(k\right)\right\}. \end{array}$$

Thus, we can obtain the following:

(17) $$\begin{array}{c} E\left\{V\left(x\left(k+1\right),m_{k+1}=j\right)\right\}<\alpha E\left\{V\left(x\left(k\right),m_{k}=i\right)\right\}+\gamma^{2}E\left\{v^{T}\left(k\right)v\left(k\right)\right\}. \end{array}$$

Observing that α>1, from (17) we obtain the following:

(18) $$\begin{array}{cc} E\{V\left(x\left(k\right),m_{k}=i\right)\}< & \alpha^{k}E\left\{V\left(x\left(0\right),m_{0}\right)\right\}+\gamma^{2}\sum_{l=0}^{k-1}\alpha^{k-1-l}E\left\{v^{T}\left(l\right)v\left(l\right)\right\}\\ < & \alpha^{\hat{T}}E\left\{V\left(x\left(0\right),m_{0}\right)\right\}+\gamma^{2}\alpha^{\hat{T}}h. \end{array}$$

Letting ${\bar{P}}_{i}=R_{i}^{-\frac{1}{2}}P_{i}R_{i}^{-\frac{1}{2}},\;{\bar{M}}_{i}=R_{i}^{-\frac{1}{2}}MR_{i}^{-\frac{1}{2}}$. According to (7), we have the following:

(19) $$\begin{array}{cc} E\{V\left(x\left(0\right),m_{0}\right)\}\; & \leq \underset{i\in S}{\sup}\left\{\lambda_{max}\left({\bar{P}}_{i}\right)\right\}E\left\{x^{T}\left(0\right)R_{i}x\left(0\right)\right\}+\underset{i\in S}{\sup}\left\{\lambda_{max}\left({\bar{M}}_{i}\right)\right\}E\left\{\sum_{l=-\tau }^{-1}x^{T}\left(l\right)R_{i}x\left(l\right)\right\}\\ \; & \leq \left[\underset{i\in S}{\sup}\left\{\lambda_{max}\left({\bar{P}}_{i}\right)\right\}+\underset{i\in S}{\sup}\left\{\lambda_{max}\left({\bar{M}}_{i}\right)\right\}\tau \right]\rho_{1}, \end{array}$$

and

(20) $$\begin{array}{cc} E\{V\left(x\left(k\right),m_{k}=i\right)\} & \geq E\left\{x^{T}\left(k\right)P_{i}x\left(k\right)\right\}=E\left\{x^{T}\left(k\right)R_{i}^{\frac{1}{2}}{\bar{P}}_{i}R_{i}^{\frac{1}{2}}x\left(k\right)\right\}\\ \; & \geq \underset{i\in S}{\inf}\left\{\lambda_{min}\left({\bar{P}}_{i}\right)\right\}E\left\{x^{T}\left(k\right)R_{i}x\left(k\right)\right\}. \end{array}$$

Combining with (18)–(20), it can be inferred that:

(21) $$E\left\{x^{T}\left(k\right)R_{i}x\left(k\right)\right\}<\frac{\alpha^{\hat{T}}\rho_{1}\left[\sup_{i\in S}\left\{\lambda_{max}\left({\bar{P}}_{i}\right)\right\}+\sup_{i\in S}\left\{\lambda_{max}\left({\bar{M}}_{i}\right)\right\}\tau \right]+\gamma^{2}\alpha^{\hat{T}}h}{\inf_{i\in S}\left\{\lambda_{min}\left({\bar{P}}_{i}\right)\right\}}.$$

Together with (12) and (21), it is clear that $E\left\{x^{T}\left(k\right)R_{i}x\left(k\right)\right\}<\rho_{2},\;k\in \left\{0,1,2,\cdots ,\hat{T}\right\}$. This implies that the CLS (9) satisfies SFT boundedness. Next, we demonstrate that the $H_{\infty }$ condition (10) holds under the zero initial condition. From (13), we can obtain the following:

(22) $$\begin{array}{c} E\left\{V\left(x\left(k+1\right),m_{k+1}=j\right)\right\}<\alpha E\left\{V\left(x\left(k\right),m_{k}=i\right)\right\}-E\left\{z^{T}\left(k\right)z\left(k\right)\right\}+\gamma^{2}E\left\{v^{T}\left(k\right)v\left(k\right)\right\}. \end{array}$$

Then, we have the following:

(23) $$\begin{array}{cc} E\{V\left(x\left(k\right),m_{k}=i\right)\}< & \alpha^{k}E\left\{V\left(x\left(0\right),m_{0}\right)\right\}-\sum_{l=0}^{k-1}\alpha^{k-1-l}E\left\{z^{T}\left(l\right)z\left(l\right)\right\}\\ + & \gamma^{2}E\left\{\sum_{l=0}^{k-1}\alpha^{k-1-l}v^{T}\left(l\right)v\left(l\right)\right\}. \end{array}$$

Assuming a zero initial condition and recognizing that $V(x\left(k\right),m_{k}=i)\geq 0$ for all $k\in \{0,1,2,\cdots ,\hat{T}\}$, we have the following:

(24) $$\sum_{l=0}^{k-1}\alpha^{k-1-l}E\left\{z^{T}\left(l\right)z\left(l\right)\right\}<\gamma^{2}E\left\{\sum_{l=0}^{k-1}\alpha^{k-1-l}v^{T}\left(l\right)v\left(l\right)\right\}.$$

Noting that α>1, from (24) we obtain the following:

(25) $$\begin{array}{c} E\left\{\sum_{k=0}^{\hat{T}}z^{T}\left(k\right)z\left(k\right)\right\}<\gamma^{2}E\left\{\sum_{k=0}^{\hat{T}}v^{T}\left(k\right)v\left(k\right)\right\}. \end{array}$$

Therefore the closed-loop MJS (9) is SFT $H_{\infty }$-bounded. □

**Remark** **3.** *It is important to note that Theorem 1 is preliminary, and since it does not provide a way to choose $K_{i}$, one can check (11), (12) on the closed-loop matrices, but this requires that $K_{i}$ has already been chosen. The problem is solved in Theorem 2.*

**Theorem** **2.** *Consider the state feedback controller (8); if there exist scalars $\alpha >1,\;\gamma >0,\;\rho_{2}>0,\;\sigma_{1}>0,\;\xi_{1}>0,\;\xi_{2}>0$ and matrices J>0, $X_{i}>0$, and $Y_{i}$, for all i∈S, satisfying the following conditions:*

(26) $$\left[\begin{array}{cccccc} -\alpha X_{i} & 0 & \Omega_{{\bar{A}}_{1i}}^{T} & \Omega_{{\bar{A}}_{2i}}^{T} & {\bar{D}}_{1i}^{T} & X_{i}\\ {}* & -\gamma^{2}I & \Omega_{C_{1i}}^{T} & \Omega_{C_{2i}}^{T} & G_{2i}^{T} & 0\\ {}* & * & -X+\Omega_{A_{d1i}}J\Omega_{A_{d1i}}^{T} & 0 & 0 & 0\\ {}* & * & * & -X+\Omega_{A_{d2i}}J\Omega_{A_{d2i}}^{T} & 0 & 0\\ {}* & * & * & * & -I+D_{d1i}JD_{d1i}^{T} & 0\\ {}* & * & * & * & * & -J \end{array}\right]<0,$$

(27) $$\sigma_{1}R_{i}^{-1}<X_{i}<R_{i}^{-1},$$

(28) $$\xi_{1}R_{i}^{-1}<J<\xi_{2}R_{i}^{-1},$$

(29) $$\left[\begin{array}{ccc} -\alpha^{-\hat{T}}\rho_{2}+\gamma^{2}h & \sqrt{\rho_{1}} & \sqrt{\tau \rho_{1}}\\ {}* & -\sigma_{1} & 0\\ {}* & * & -\xi_{1} \end{array}\right]<0,$$

*where* *$\Omega_{{\bar{A}}_{1i}}^{T}=[\sqrt{\pi_{i\zeta_{1}}}{\left(A_{1i}X_{i}+B_{1i}Y_{i}\right)}^{T}\;\sqrt{\pi_{i\zeta_{2}}}{\left(A_{1i}X_{i}+B_{1i}Y_{i}\right)}^{T}\;\cdots \;\sqrt{\pi_{i\zeta_{p_{i}}}}{\left(A_{1i}X_{i}+B_{1i}Y_{i}\right)}^{T}\;\;\;\;\;\;\;\sqrt{1-\pi_{k}^{i}}{\left(A_{1i}X_{i}+B_{1i}Y_{i}\right)}^{T}\;\sqrt{1-\pi_{k}^{i}}{\left(A_{1i}X_{i}+B_{1i}Y_{i}\right)}^{T}\;\cdots \;\sqrt{1-\pi_{k}^{i}}{\left(A_{1i}X_{i}+B_{1i}Y_{i}\right)}^{T}],$
*

       *$\Omega_{{\bar{A}}_{2i}}^{T}=[\sqrt{\pi_{i\zeta_{1}}}{\left(A_{2i}X_{i}+B_{2i}Y_{i}\right)}^{T}\;\sqrt{\pi_{i\zeta_{2}}}{\left(A_{2i}X_{i}+B_{2i}Y_{i}\right)}^{T}\;\cdots \;\sqrt{\pi_{i\zeta_{p_{i}}}}{\left(A_{2i}X_{i}+B_{2i}Y_{i}\right)}^{T}\;\;\;\;\;\;\;\sqrt{1-\pi_{k}^{i}}{\left(A_{2i}X_{i}+B_{2i}Y_{i}\right)}^{T}\;\sqrt{1-\pi_{k}^{i}}{\left(A_{2i}X_{i}+B_{2i}Y_{i}\right)}^{T}\;\cdots \;\sqrt{1-\pi_{k}^{i}}{\left(A_{2i}X_{i}+B_{2i}Y_{i}\right)}^{T}],$*

       *$\Omega_{A_{d1i}}^{T}=\left[\sqrt{\pi_{i\zeta_{1}}}A_{d1i}^{T}\;\sqrt{\pi_{i\zeta_{2}}}A_{d1i}^{T}\;\cdots \;\sqrt{\pi_{i\zeta_{p_{i}}}}A_{d1i}^{T}\;\sqrt{1-\pi_{k}^{i}}A_{d1i}^{T}\;\sqrt{1-\pi_{k}^{i}}A_{d1i}^{T}\;\cdots \;\sqrt{1-\pi_{k}^{i}}A_{d1i}^{T}\right]$,*

       *$\Omega_{A_{d2i}}^{T}=\left[\sqrt{\pi_{i\zeta_{1}}}A_{d2i}^{T}\;\sqrt{\pi_{i\zeta_{2}}}A_{d2i}^{T}\;\cdots \;\sqrt{\pi_{i\zeta_{p_{i}}}}A_{d2i}^{T}\;\sqrt{1-\pi_{k}^{i}}A_{d2i}^{T}\;\sqrt{1-\pi_{k}^{i}}A_{d2i}^{T}\;\cdots \;\sqrt{1-\pi_{k}^{i}}A_{d2i}^{T}\right],$*

       *$\Omega_{C_{1i}}^{T}=\left[\sqrt{\pi_{i\zeta_{1}}}C_{1i}^{T}\;\sqrt{\pi_{i\zeta_{2}}}C_{1i}^{T}\;\cdots \;\sqrt{\pi_{i\zeta_{p_{i}}}}C_{1i}^{T}\sqrt{1-\pi_{k}^{i}}C_{1i}^{T}\;\sqrt{1-\pi_{k}^{i}}C_{1i}^{T}\;\cdots \;\sqrt{1-\pi_{k}^{i}}C_{1i}^{T}\right]$,*

       *$\Omega_{C_{2i}}^{T}=\left[\sqrt{\pi_{i\zeta_{1}}}C_{2i}^{T}\;\sqrt{\pi_{i\zeta_{2}}}C_{2i}^{T}\;\cdots \;\sqrt{\pi_{i\zeta_{p_{i}}}}C_{2i}^{T}\;\sqrt{1-\pi_{k}^{i}}C_{2i}^{T}\;\sqrt{1-\pi_{k}^{i}}C_{2i}^{T}\;\cdots \;\sqrt{1-\pi_{k}^{i}}C_{2i}^{T}\right],$*

       *$X=diag\{X_{\zeta_{1}},X_{\zeta_{2}},\cdots ,X_{\zeta_{p_{i}}},X_{\zeta_{u1}},X_{\zeta_{u2}},\cdots ,X_{\zeta_{uq_{i}}}\}$,  ${\bar{D}}_{1i}=D_{1i}X_{i}+G_{1i}Y_{i}$,*

*then the CLS (9) with the partly unknown TPs is SFT $H_{\infty }$-bounded with respect to $(\rho_{1}$,$\rho_{2}$,$R_{i}$,$\hat{T}$,γ,h), i.e., MJS (1) is SFT $H_{\infty }$ state feedback stabilization, and the controller gain $K_{i}=Y_{i}X_{i}^{-1}$.*

**Proof.**  First, we demonstrate the equivalence between condition (26) and condition (11). According to Lemma 1, (26) is equivalent to the following:

(30) $$\left[\begin{array}{cccccc} -\alpha X_{i}+X_{i}J^{-1}X_{i} & 0 & 0 & \Omega_{{\bar{A}}_{1i}}^{T} & \Omega_{{\bar{A}}_{2i}}^{T} & {\bar{D}}_{1i}^{T}\\ {}* & -J^{-1} & 0 & \Omega_{A_{d1i}}^{T} & \Omega_{A_{d2i}}^{T} & D_{d1i}^{T}\\ {}* & * & -\gamma^{2}I & \Omega_{C_{1i}}^{T} & \Omega_{C_{2i}}^{T} & G_{2i}^{T}\\ {}* & * & * & -X & 0 & 0\\ {}* & * & * & * & -X & 0\\ {}* & * & * & * & * & -I \end{array}\right]<0.$$

Letting $X_{i}=P_{i}^{-1},\;J=M^{-1},\;X=P^{-1},\;K_{i}=Y_{i}X_{i}^{-1}$. Pre- and post-multiplying (30) by $diag\{X_{i}^{-1},I,I,I,I,I\}$, we can observe that (30) is equivalent to (11) by using Lemma 1.

On the other hand, from Lemma 1, (29) can be expressed as the following inequality:

(31) $$\alpha^{\hat{T}}\rho_{1}\left(\sigma_{1}^{-1}+\xi_{1}^{-1}\tau \right)+\gamma^{2}\alpha^{\hat{T}}h<\rho_{2}.$$

We note that ${\bar{P}}_{i}=R_{i}^{-\frac{1}{2}}P_{i}R_{i}^{-\frac{1}{2}}$ and ${\bar{M}}_{i}=R_{i}^{-\frac{1}{2}}MR_{i}^{-\frac{1}{2}}$; combined with conditions (27) and (28), we can obtain that:

$$\underset{i\in S}{\sup}\left\{\lambda_{max}\left({\bar{P}}_{i}\right)\right\}<\sigma_{1}^{-1},\;\underset{i\in S}{\inf}\left\{\lambda_{min}\left({\bar{P}}_{i}\right)\right\}>1,\;\underset{i\in S}{\sup}\left\{\lambda_{max}\left({\bar{M}}_{i}\right)\right\}<\xi_{1}^{-1}.$$

Therefore, it is easy to observe that (12) holds. This completes the proof. □

**Remark** **4.** *Theorem 2 generalizes the results of [17] to a time-delay MJS, and gives sufficient conditions for MJS (1) to be SFT $H_{\infty }$ state feedback stabilization.*

**Remark** **5.** *It can be seen from Theorem 2 that the controller gain $K_{i}$ depends on $X_{i}$ and $Y_{i}$, that in turn depend on the system matrices of mode i. It is necessary to consider the dynamic characteristics of the system in different modes and the transition probabilities between modes to ensure the stability and the controller in each mode.*

## 4. Finite-Time $H_{\infty }$ Observer-Based State Feedback Control

### 4.1. Observer-Based State Feedback Controller

In the presence of a system state that is not fully measurable, the following observer-based state feedback controller is designed:

(32) $$\left\{\begin{array}{cc} \hat{x}\left(k+1\right)\;=\; & A_{1}\left(m_{k}\right)\hat{x}\left(k\right)+A_{d1}\left(m_{k}\right)\hat{x}\left(k-\tau \right)+B_{1}\left(m_{k}\right)u\left(k\right)+H\left(m_{k}\right)\left[y\left(k\right)-\hat{y}\left(k\right)\right],\\ \hat{y}\left(k\right)\;=\; & D\left(m_{k}\right)\hat{x}\left(k\right)+G\left(m_{k}\right)u\left(k\right),\\ u(k)\;=\; & \hat{K}\left(m_{k}\right)\hat{x}\left(k\right),\\ \hat{x}\left(n\right)\;=\; & \psi (n),\;\;\;\;n\in \{-\tau ,-\tau +1,\cdots ,0\}, \end{array}\right.$$

where $\hat{x}\left(k\right)$ is the estimated state and $\hat{y}\left(k\right)$ is the estimated output, and $\hat{K}\left(m_{k}\right)$ and $H(m_{k})$ denote the state feedback gain and observer gain to be determined, respectively. The estimated state error is defined as $e\left(k\right)=x\left(k\right)-\hat{x}\left(k\right)$, and $\eta^{T}\left(k\right)=\left[\;x^{T}\left(k\right)\;\;e^{T}\left(k\right)\;\right]$. For $m_{k}=i\;\left(i\in S\right)$, the CLS is represented by the following:

(33) $$\left\{\begin{array}{cc} \eta (k+1)\;=\; & {\hat{A}}_{1i}\eta \left(k\right)+{\hat{A}}_{d1i}\eta \left(k-\tau \right)+{\hat{C}}_{1i}v\left(k\right)\\ \; & +[{\hat{A}}_{2i}\eta \left(k\right)+{\hat{A}}_{d2i}\eta \left(k-\tau \right)+{\hat{C}}_{2i}v\left(k\right)]\omega \left(k\right),\\ z(k)\;=\; & {\hat{D}}_{1i}\eta \left(k\right)+{\hat{D}}_{d1i}\eta \left(k-\tau \right)+G_{2i}v\left(k\right), \end{array}\right.$$

where ${\hat{A}}_{1i}=\left[\begin{array}{cc} A_{1i}+B_{1i}{\hat{K}}_{i} & -B_{1i}{\hat{K}}_{i}\\ 0 & A_{1i}-H_{i}D_{i} \end{array}\right]$,  ${\hat{A}}_{d1i}=\left[\begin{array}{cc} A_{d1i} & 0\\ 0 & A_{d1i} \end{array}\right]$,  ${\hat{C}}_{1i}=\left[\begin{array}{c} C_{1i}\\ C_{1i} \end{array}\right]$,

       ${\hat{A}}_{2i}=\left[\begin{array}{cc} A_{2i}+B_{2i}{\hat{K}}_{i} & -B_{2i}{\hat{K}}_{i}\\ A_{2i}+B_{2i}{\hat{K}}_{i} & -B_{2i}{\hat{K}}_{i} \end{array}\right]$,  ${\hat{A}}_{d2i}=\left[\begin{array}{cc} A_{d2i} & 0\\ A_{d2i} & 0 \end{array}\right]$,  ${\hat{C}}_{2i}=\left[\begin{array}{c} C_{2i}\\ C_{2i} \end{array}\right]$,

       ${\hat{D}}_{1i}=\left[\begin{array}{cc} D_{1i}+G_{1i}{\hat{K}}_{i} & -G_{1i}{\hat{K}}_{i} \end{array}\right],\;\;\;{\hat{D}}_{d1i}=\left[\begin{array}{cc} D_{d1i} & 0 \end{array}\right].$

**Definition** **5.** *(SFT $H_{\infty }$ boundedness)*

*The CLS (33) is said to be SFT $H_{\infty }$-bounded with respect to $\left(\rho_{1},\rho_{2},{\hat{R}}_{i},\hat{T},h,\gamma \right)$ if the following two conditions hold:*

*(a)*  *The MJS (33) satisfies SFT boundedness with respect to $\left(\rho_{1},\rho_{2},{\hat{R}}_{i},\hat{T},h\right)$;*
*(b)*  *Under the zero initial condition, for the external disturbance v(k) satisfying condition (2), the control output z(k) satisfies the following:*

(34) $$E\left\{\sum_{k=0}^{\hat{T}}z^{T}\left(k\right)z\left(k\right)\right\}<\gamma^{2}E\left\{\sum_{k=0}^{\hat{T}}v^{T}\left(k\right)v\left(k\right)\right\},$$

*where ${\hat{R}}_{i}>0,\;i\in S$, $0<\rho_{1}<\rho_{2}$, γ>0.*

**Definition** **6.** *(SFT $H_{\infty }$ observer-based state feedback stabilization)*

*The MJS (1) is said to be SFT $H_{\infty }$ observer-based state feedback stabilization with respect to $\left(\rho_{1},\rho_{2},{\hat{R}}_{i},\hat{T},h,\gamma \right)$ if there exists an observer-based state feedback controller (32) such that the CLS (33) satisfies SFT $H_{\infty }$ boundedness, and the controller (32) is called the SFT $H_{\infty }$ observer-based state feedback controller.*

### 4.2. Main Results

**Theorem** **3.** *The CLS (33) with the partly unknown TPs is SFT $H_{\infty }$-bounded with respect to $\left(\rho_{1},\rho_{2},{\hat{R}}_{i},\hat{T},\gamma ,h\right)$ if there exist the scalars β>1 and γ>0, matrix $\hat{M}>0$, and positive-definite matrices ${\hat{P}}_{i}$ for all i∈S, satisfying the following conditions:*

(35) $$\left[\begin{array}{ccc} -\beta {\hat{P}}_{i}+\hat{M}+\Gamma_{1i}^{T}+{\hat{D}}_{1i}^{T}{\hat{D}}_{1i} & \Gamma_{2i}^{T}+{\hat{D}}_{1i}^{T}{\hat{D}}_{d1i} & \Gamma_{3i}^{T}+{\hat{D}}_{1i}^{T}G_{2i}\;\\ \;* & \Gamma_{4i}^{T}-\hat{M}+{\hat{D}}_{d1i}^{T}{\hat{D}}_{d1i} & \Gamma_{5i}^{T}+{\hat{D}}_{d1i}^{T}G_{2i}\\ {}* & * & \Gamma_{6i}^{T}-\gamma^{2}+G_{2i}^{T}G_{2i} \end{array}\right]<0,$$

(36) $$\beta^{\hat{T}}\rho_{1}\left[\underset{i\in S}{\sup}\left\{\lambda_{max}\left({\tilde{P}}_{i}\right)\right\}+\underset{i\in S}{\sup}\left\{\lambda_{max}\left({\tilde{M}}_{i}\right)\right\}\tau \right]+\gamma^{2}\beta^{\hat{T}}h<\underset{i\in S}{\inf}\left\{\lambda_{min}\left({\tilde{P}}_{i}\right)\right\}\rho_{2},$$

*where* ${\hat{P}}_{i}=diag\left\{P_{i},P_{i}\right\},\;\hat{M}=diag\left\{M,M\right\},\;{\tilde{P}}_{i}={\hat{R}}_{i}^{-\frac{1}{2}}{\hat{P}}_{i}{\hat{R}}_{i}^{-\frac{1}{2}},\;{\tilde{M}}_{i}={\hat{R}}_{i}^{-\frac{1}{2}}\hat{M}{\hat{R}}_{i}^{-\frac{1}{2}},$

        *$\Gamma_{1i}^{T}={\hat{A}}_{1i}^{T}{\hat{\Psi }}_{i}{\hat{A}}_{1i}+{\hat{A}}_{2i}^{T}{\hat{\Psi }}_{i}{\hat{A}}_{2i},\;\;\Gamma_{2i}^{T}={\hat{A}}_{1i}^{T}{\hat{\Psi }}_{i}{\hat{A}}_{d1i}+{\hat{A}}_{2i}^{T}{\hat{\Psi }}_{i}{\hat{A}}_{d2i},\;\;\Gamma_{3i}^{T}={\hat{A}}_{1i}^{T}{\hat{\Psi }}_{i}{\hat{C}}_{1i}+{\hat{A}}_{2i}^{T}{\hat{\Psi }}_{i}{\hat{C}}_{2i},$*

        *$\Gamma_{4i}^{T}={\hat{A}}_{d1i}^{T}{\hat{\Psi }}_{i}{\hat{A}}_{d1i}+{\hat{A}}_{d2i}^{T}{\hat{\Psi }}_{i}{\hat{A}}_{d2i},\;\;\Gamma_{5i}^{T}={\hat{A}}_{d1i}^{T}{\hat{\Psi }}_{i}{\hat{C}}_{1i}+{\hat{A}}_{d2i}^{T}{\hat{\Psi }}_{i}{\hat{C}}_{2i},\;\;\Gamma_{6i}^{T}={\hat{C}}_{1i}^{T}{\hat{\Psi }}_{i}{\hat{C}}_{1i}+{\hat{C}}_{2i}^{T}{\hat{\Psi }}_{i}{\hat{C}}_{2i},$*

        *${\hat{\Psi }}_{i}=\sum_{j\in S_{k}^{i}}\pi_{ij}{\hat{P}}_{j}+\left(1-\pi_{k}^{i}\right)\left(\sum_{j\in S_{uk}^{i}}{\hat{P}}_{j}\right).$*

**Proof.**  The proof procedure is similar to Theorem 1, and thus will not be reiterated. □

From the above discussion, system (33) is SFT $H_{\infty }$-bounded. Then, the following theorem will develop the observer-based state feedback controller for system (33).

**Theorem** **4.** *The CLS (33) with the partly unknown TPs is SFT $H_{\infty }$-bounded with respect to $\left(\rho_{1},\rho_{2},{\hat{R}}_{i},\hat{T},\gamma ,h\right)$ if there exist the scalars $\beta >1,\;\gamma >0,\;\rho_{2}>0,\;\sigma_{2}>0,\;\varrho_{1}>0,\;\varrho_{2}>0$, matrix J>0, positive-definite matrices $X_{i}$, nonsingular matrices $Z_{i}$, and matrices $Y_{i}$ and $F_{i}$ for all i∈S, satisfying the following conditions:*

(37) $$\left[\begin{array}{cccccc} -\beta {\hat{X}}_{i} & 0 & \Xi_{{\tilde{A}}_{1i}}^{T} & \Xi_{{\tilde{A}}_{2i}}^{T} & {\tilde{D}}_{1i}^{T} & {\hat{X}}_{i}\\ {}* & -\gamma^{2}I & \Xi_{{\hat{C}}_{1i}}^{T} & \Xi_{{\hat{C}}_{2i}}^{T} & G_{2i}^{T} & 0\\ {}* & * & -\hat{X}+\Xi_{{\hat{A}}_{d1i}}\hat{J}\Xi_{{\hat{A}}_{d1i}}^{T} & 0 & 0 & 0\\ {}* & * & * & -\hat{X}+\Xi_{{\hat{A}}_{d2i}}\hat{J}\Xi_{{\hat{A}}_{d2i}}^{T} & 0 & 0\\ {}* & * & * & * & -I+{\hat{D}}_{d1i}\hat{J}{\hat{D}}_{d1i}^{T} & 0\\ {}* & * & * & * & * & -\hat{J} \end{array}\right]<0,$$

(38) $$\begin{array}{c} D_{i}X_{i}=Z_{i}D_{i}, \end{array}$$

(39) $$\sigma_{2}{\hat{R}}_{i}^{-1}<{\hat{X}}_{i}<{\hat{R}}_{i}^{-1},$$

(40) $$\varrho_{1}{\hat{R}}_{i}^{-1}<\hat{J}<\varrho_{2}{\hat{R}}_{i}^{-1},$$

(41) $$\left[\begin{array}{ccc} -\beta^{-\hat{T}}\rho_{2}+\gamma^{2}h & \sqrt{\rho_{1}} & \sqrt{\tau \rho_{1}}\\ {}* & -\sigma_{2} & 0\\ {}* & * & -\varrho_{1} \end{array}\right]<0,$$

*where* *$\Xi_{{\tilde{A}}_{1i}}^{T}=\left[\sqrt{\pi_{i\zeta_{1}}}{\tilde{A}}_{1i}^{T}\;\sqrt{\pi_{i\zeta_{2}}}{\tilde{A}}_{1i}^{T}\;\cdots \;\sqrt{\pi_{i\zeta_{p_{i}}}}{\tilde{A}}_{1i}^{T}\;\sqrt{1-\pi_{k}^{i}}{\tilde{A}}_{1i}^{T}\;\sqrt{1-\pi_{k}^{i}}{\tilde{A}}_{1i}^{T}\;\cdots \;\sqrt{1-\pi_{k}^{i}}{\tilde{A}}_{1i}^{T}\right]$,*

    *$\Xi_{{\tilde{A}}_{2i}}^{T}=\left[\sqrt{\pi_{i\zeta_{1}}}{\tilde{A}}_{2i}^{T}\;\sqrt{\pi_{i\zeta_{2}}}{\tilde{A}}_{2i}^{T}\;\cdots \;\sqrt{\pi_{i\zeta_{p_{i}}}}{\tilde{A}}_{2i}^{T}\;\sqrt{1-\pi_{k}^{i}}{\tilde{A}}_{2i}^{T}\;\sqrt{1-\pi_{k}^{i}}{\tilde{A}}_{2i}^{T}\;\cdots \;\sqrt{1-\pi_{k}^{i}}{\tilde{A}}_{2i}^{T}\right],$*

    *${\tilde{A}}_{1i}=\left[\begin{array}{cc} A_{1i}X_{i}+B_{1i}Y_{i} & -B_{1i}Y_{i}\\ 0 & A_{1i}X_{i}-F_{i}D_{i} \end{array}\right],$ ${\tilde{A}}_{2i}=\left[\begin{array}{cc} A_{2i}X_{i}+B_{2i}Y_{i} & -B_{2i}Y_{i}\\ A_{2i}X_{i}+B_{2i}Y_{i} & -B_{2i}Y_{i} \end{array}\right],$*

    *$\Xi_{{\hat{A}}_{d1i}}^{T}=\left[\sqrt{\pi_{i\zeta_{1}}}{\hat{A}}_{d1i}^{T}\;\sqrt{\pi_{i\zeta_{2}}}{\hat{A}}_{d1i}^{T}\;\cdots \;\sqrt{\pi_{i\zeta_{p_{i}}}}{\hat{A}}_{d1i}^{T}\;\sqrt{1-\pi_{k}^{i}}{\hat{A}}_{d1i}^{T}\;\sqrt{1-\pi_{k}^{i}}{\hat{A}}_{d1i}^{T}\;\cdots \;\sqrt{1-\pi_{k}^{i}}{\hat{A}}_{d1i}^{T}\right]$,*

    *$\Xi_{{\hat{A}}_{d2i}}^{T}=\left[\sqrt{\pi_{i\zeta_{1}}}{\hat{A}}_{d2i}^{T}\;\sqrt{\pi_{i\zeta_{2}}}{\hat{A}}_{d2i}^{T}\;\cdots \;\sqrt{\pi_{i\zeta_{p_{i}}}}{\hat{A}}_{d2i}^{T}\;\sqrt{1-\pi_{k}^{i}}{\hat{A}}_{d2i}^{T}\;\sqrt{1-\pi_{k}^{i}}{\hat{A}}_{d2i}^{T}\;\cdots \;\sqrt{1-\pi_{k}^{i}}{\hat{A}}_{d2i}^{T}\right],$*

    *$\Xi_{{\hat{C}}_{1i}}^{T}=\left[\sqrt{\pi_{i\zeta_{1}}}{\hat{C}}_{1i}^{T}\;\sqrt{\pi_{i\zeta_{2}}}{\hat{C}}_{1i}^{T}\;\cdots \;\sqrt{\pi_{i\zeta_{p_{i}}}}{\hat{C}}_{1i}^{T}\;\sqrt{1-\pi_{k}^{i}}{\hat{C}}_{1i}^{T}\;\sqrt{1-\pi_{k}^{i}}{\hat{C}}_{1i}^{T}\;\cdots \;\sqrt{1-\pi_{k}^{i}}{\hat{C}}_{1i}^{T}\right]$,*

    *$\Xi_{{\hat{C}}_{2i}}^{T}=\left[\sqrt{\pi_{i\zeta_{1}}}{\hat{C}}_{2i}^{T}\;\sqrt{\pi_{i\zeta_{2}}}{\hat{C}}_{2i}^{T}\;\cdots \;\sqrt{\pi_{i\zeta_{p_{i}}}}{\hat{C}}_{2i}^{T}\;\sqrt{1-\pi_{k}^{i}}{\hat{C}}_{2i}^{T}\;\sqrt{1-\pi_{k}^{i}}{\hat{C}}_{2i}^{T}\;\cdots \;\sqrt{1-\pi_{k}^{i}}{\hat{C}}_{2i}^{T}\right],$*

    *${\tilde{D}}_{1i}=\left[\;D_{1i}X_{i}+G_{1i}Y_{i}\;\;-G_{1i}Y_{i}\;\right],\;\;{\hat{X}}_{i}=diag\left\{X_{i},X_{i}\right\},\;\;\hat{J}=diag\left\{J,J\right\},\;\;{\hat{R}}_{i}=diag\left\{R_{i},R_{i}\right\}$,*

    *$\hat{X}=diag\left\{{\hat{X}}_{\zeta_{1}},{\hat{X}}_{\zeta_{2}},\cdots ,{\hat{X}}_{\zeta_{p_{i}}},{\hat{X}}_{\zeta_{u1}},{\hat{X}}_{\zeta_{u2}},\cdots ,{\hat{X}}_{\zeta_{uq_{i}}}\right\}$.*

*Then, MJS (1) is called SFT $H_{\infty }$ observer-based state feedback stabilization, and the controller gain ${\hat{K}}_{i}$ as well as the observer gain $H_{i}$ are represented as follows:*

(42) $${\hat{K}}_{i}=Y_{i}X_{i}^{-1},\;H_{i}=F_{i}Z_{i}^{-1}.$$

**Proof.**  Defining ${\hat{R}}_{i}=diag\left\{R_{i},R_{i}\right\},\;{\hat{P}}_{i}=diag\left\{P_{i},P_{i}\right\}$, $X_{i}=P_{i}^{-1},\;\hat{X}={\hat{P}}^{-1},\hat{M}=diag\left\{M,M\right\},$ $M^{-1}=J,\;{\hat{K}}_{i}=Y_{i}X_{i}^{-1},\;H_{i}=F_{i}Z_{i}^{-1},$ and taking into account condition (38), (35) will be obtained from (37) via Lemma 1. In addition, we denote ${\tilde{P}}_{i}={\hat{R}}_{i}^{-\frac{1}{2}}{\hat{P}}_{i}{\hat{R}}_{i}^{-\frac{1}{2}},\;{\tilde{M}}_{i}={\hat{R}}_{i}^{-\frac{1}{2}}\hat{M}{\hat{R}}_{i}^{-\frac{1}{2}}$. According to the proof of Theorem 2, it is obvious that condition (36) will be guaranteed by (39) to (41). □

**Remark** **6.** *Theorems 3 and 4 extend the results of [38,44] to MJSs with partly unknown TPs.*

**Remark** **7.** *Addressing condition (38) through the application of the LMI toolbox is a challenging task. As a solution, constraint (38) can be approximated by the following inequality:*

(43) $${\left[D_{i}X_{i}-Z_{i}D_{i}\right]}^{T}\left[D_{i}X_{i}-Z_{i}D_{i}\right]<\varpi I,$$

*where ϖ represents an exceedingly small positive scalar. According to Lemma 1, the above inequality can be formulated as follows:*

(44) $$\left[\begin{array}{cc} -\varpi I & {\left[D_{i}X_{i}-Z_{i}D_{i}\right]}^{T}\\ {}* & -I \end{array}\right]<0.$$

**Remark** **8.** *We can note that conditions (26), (29), (37), and (41) are not strict LMIs; however, once we fix the parameters α and β, the conditions can be turned into LMI-based feasibility problems. Therefore, the feasibility of the conditions stated in Theorems 2 and 4 can be turned into the following feasibility problems with the fixed parameters α and β, respectively:*

(45) $$\begin{array}{cc} \; & min\;(\rho_{2}+\gamma^{2})\\ \; & s.t.\;LMIs\;(26),\;(27),\;(28)\;and\;(29), \end{array}$$

(46) $$\begin{array}{cc} \; & min\;(\rho_{2}+\gamma^{2})\\ \; & s.t.\;LMIs\;(37),\;(39),\;(40),\;(41)\;and\;(44). \end{array}$$

## 5. Numerical Examples

In this section, we present two examples to validate the effectiveness and practicality of the proposed method. The first example is used to show the effectiveness of the state feedback controller (8) design approach developed in Theorem 2 for MJSs (1) with partly unknown transition probabilities.

**Example** **1.** *Consider MJS (1) with three modes, and the coefficient matrices are given as follows:*

*Mode 1 (i=1):*

*$A_{11}=\left[\begin{array}{cc} 0.1 & 0\\ 0 & 0.1 \end{array}\right],$ $A_{d11}=\left[\begin{array}{cc} 0.8 & 0\\ -0.2 & 0.1 \end{array}\right],$ $B_{11}=\left[\begin{array}{c} 1\\ -0.1 \end{array}\right],$ $C_{11}=\left[\begin{array}{c} 0.1\\ 0.1 \end{array}\right],$ $A_{21}=\left[\begin{array}{cc} -0.1 & 0\\ 0 & -0.1 \end{array}\right],$ $A_{d21}=$ $\left[\begin{array}{cc} -0.1 & 1\\ 0 & 0.1 \end{array}\right],$ $B_{21}=$ $\left[\begin{array}{c} -0.1\\ 0.3 \end{array}\right],$ $C_{21}=$ $\left[\begin{array}{c} 0.1\\ 0.1 \end{array}\right],$ $D_{11}=$ $\left[\begin{array}{cc} -0.2 & 1 \end{array}\right],$ $D_{d11}=\left[\begin{array}{cc} -0.1 & 0 \end{array}\right],$ $D_{1}=\left[\begin{array}{cc} 1 & 1 \end{array}\right],$ $G_{1}=1,$ $G_{11}=-1$, $G_{21}=0.1.$*

*Mode 2 (i=2):*

*$A_{12}=\left[\begin{array}{cc} 1 & 0\\ 0.3 & 1 \end{array}\right],$ $A_{d12}=\left[\begin{array}{cc} 0 & 0.1\\ 0.1 & -0.1 \end{array}\right],$ $B_{12}=\left[\begin{array}{c} 0.1\\ -0.1 \end{array}\right],$ $C_{12}=\left[\begin{array}{c} 0.1\\ 0.1 \end{array}\right],$ $A_{22}=\left[\begin{array}{cc} 0.1 & 0\\ 0 & 0.1 \end{array}\right],$ $A_{d22}=\left[\begin{array}{cc} -0.1 & 1\\ 0 & 0.1 \end{array}\right],$ $B_{22}=\left[\begin{array}{c} -0.1\\ 0.1 \end{array}\right],$ $C_{22}=\left[\begin{array}{c} 0.1\\ 0.1 \end{array}\right],$ $D_{12}=\left[\begin{array}{cc} 1 & 0.1 \end{array}\right],$ $D_{d12}=\left[\begin{array}{cc} 0.1 & 0.1 \end{array}\right],$ $D_{2}=\left[\begin{array}{cc} 2 & 1 \end{array}\right],$ $G_{2}=1,$ $G_{12}=-1$, $G_{22}=0.2.$*

*Mode 3 (i=3):*

*$A_{13}=\left[\begin{array}{cc} -1 & 0.1\\ 0 & 0.1 \end{array}\right],$ $A_{d13}=\left[\begin{array}{cc} -0.1 & 0.1\\ 0 & 0.1 \end{array}\right],$ $B_{13}=\left[\begin{array}{c} 1.1\\ -0.1 \end{array}\right],$ $C_{13}=\left[\begin{array}{c} 0.1\\ 0.1 \end{array}\right],$ $A_{23}=\left[\begin{array}{cc} 0.3 & 0\\ 0 & 0.1 \end{array}\right],$ $A_{d23}=\left[\begin{array}{cc} 0.2 & 1\\ 0 & 0.1 \end{array}\right],$ $B_{23}=\left[\begin{array}{c} -0.1\\ 0.4 \end{array}\right],$ $C_{23}=\left[\begin{array}{c} 0.1\\ 0.1 \end{array}\right],$ $D_{13}=\left[\begin{array}{cc} 0.1 & 1 \end{array}\right],$ $D_{d13}=\left[\begin{array}{cc} -0.1 & 0.1 \end{array}\right],$ $D_{3}=\left[\begin{array}{cc} 1 & 3 \end{array}\right],$ $G_{3}=1,$ $G_{13}=-1$, $G_{23}=0.1.$*

**Figure 1:**
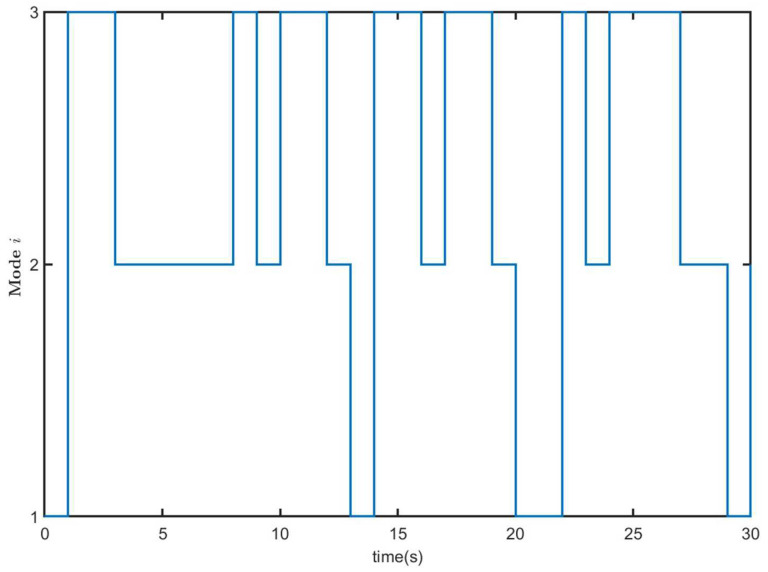
Markovian switching process of MJS (1) and CLS (9).

The partly unknown TP matrix Π with three modes is given as follows:

$$\Pi =\left[\begin{array}{ccc} {\hat{\pi }}_{11} & 0.2 & {\hat{\pi }}_{13}\\ {\hat{\pi }}_{21} & {\hat{\pi }}_{22} & 0.8\\ 0.1 & {\hat{\pi }}_{32} & {\hat{\pi }}_{33} \end{array}\right],$$

where ${\hat{\pi }}_{ij}\left(i,j=1,2,3\right)$ is the unknown element. One possible mode evolution is given in Figure 1.

**Figure 2:**
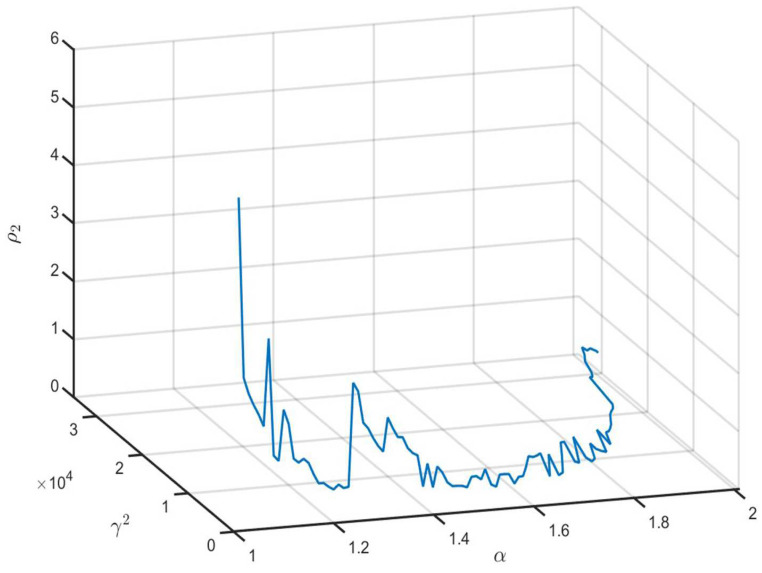
The values of $\rho_{2}$ and $\gamma^{2}$ with different α values.

**Figure 3:**
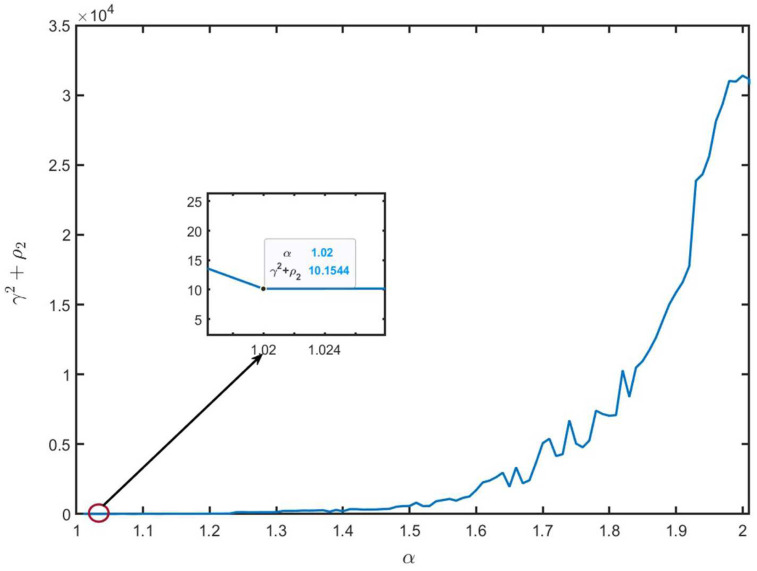
The values of $\gamma^{2}+\rho_{2}$ with different α values.

According to Remark 8, the minimum value of $\rho_{2}+\gamma^{2}$ relies on the parameter α. We can obtain the feasible solution of (45) when 1.01≤α≤2.01. Figure 2 and Figure 3 show the optimal values of $\rho_{2}$, $\gamma^{2}$ and $\rho_{2}+\gamma^{2}$ with different α values. We can see that the optimal values $\rho_{2}=2.6437$, $\gamma^{2}=7.5107$, and $\gamma^{2}+\rho_{2}=10.1544$ when α=1.02.

Next, letting $\tau =3,\;h=3,\;\hat{T}=30,\;\rho_{1}=0.1$, and $\;R_{i}=I_{2\times 2}\;\left(i=1,2,3\right)$, and solving LMIs (26) to (29), we obtain $\sigma_{1}=0.1888$, $\xi_{1}=0.3535$, and $\xi_{2}=21.5646$, and the gains of the state feedback controller (8) are as follows:

$$K_{1}=\left[\begin{array}{cc} -0.1176 & 0.2082 \end{array}\right],\;K_{2}=\left[\begin{array}{cc} 0.9916 & 0.1625 \end{array}\right],\;K_{3}=\left[\begin{array}{cc} 0.4783 & 0.3299 \end{array}\right].$$

Then, we set the initial value $x\left(0\right)={\left[\begin{array}{cc} 0 & 0 \end{array}\right]}^{T}$ for MJS (1) and CLS (9), and the external disturbance signal v(k)=0.4sink, which satisfies $\sum_{k=0}^{\hat{T}}v^{T}\left(k\right)v\left(k\right)\leq h=3$.

**Figure 4:**
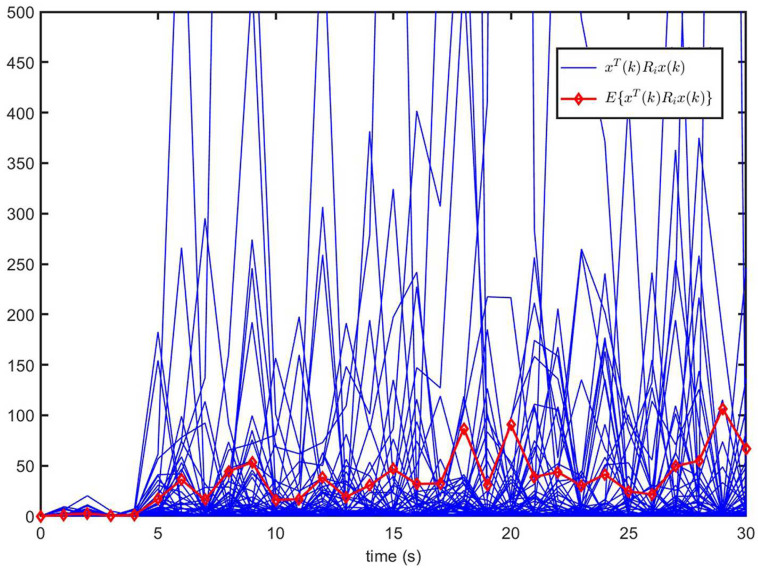
The trajectories of $x^{T}\left(k\right)R_{i}x\left(k\right)$ and $E\{x^{T}\left(k\right)R_{i}x\left(k\right)\}$ for open-loop system (1) (u(k)=0).

Figure 4 shows the trajectories of $x^{T}\left(k\right)R_{i}x\left(k\right)$ (50 curves) and $E\{x^{T}\left(k\right)R_{i}x\left(k\right)\}$ of the open-loop system (1) (u(k)=0). It can be seen that the trajectory of $E\{x^{T}\left(k\right)R_{i}x\left(k\right)\}$ exceeds the upper bound $\rho_{2}$, despite $E\left\{x^{T}\left(0\right)R_{i}x\left(0\right)\right\}=0<\rho_{1}=0.1$. This implies that the open-loop system (1) is not finite-time bound.

**Figure 5:**
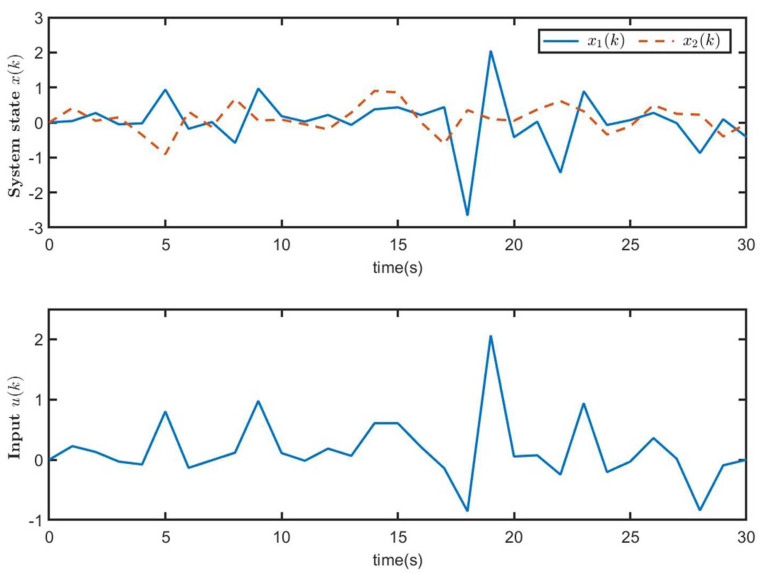
The trajectories of system state x(k) for CLS (9) and control input u(k) (8).

**Figure 6:**
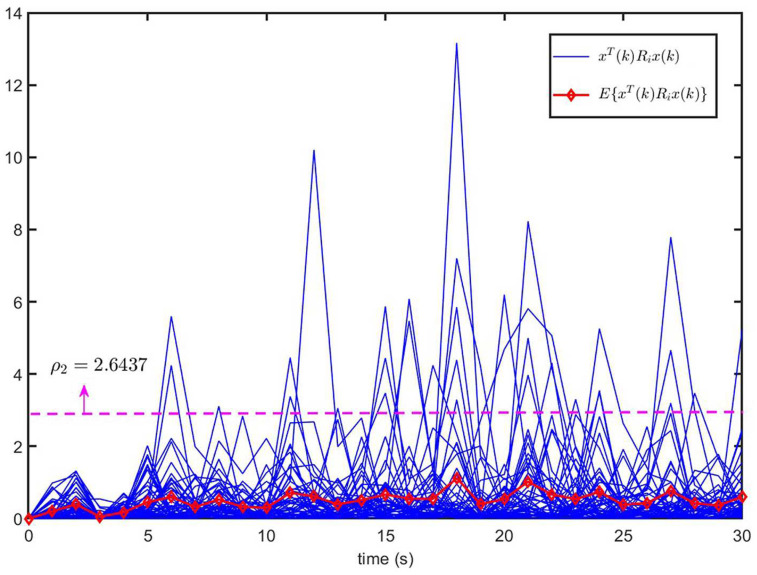
The trajectories of $x^{T}\left(k\right)R_{i}x\left(k\right)$ and $E\{x^{T}\left(k\right)R_{i}x\left(k\right)\}$ for MJS (1) and CLS (9).

Figure 5 shows the trajectories of the system state x(k) for closed-loop system (9) and the control input u(k) of MJS (1). The trajectories of $x^{T}\left(k\right)R_{i}x\left(k\right)$ (50 curves) and $E\{x^{T}\left(k\right)R_{i}x\left(k\right)\}$ of closed-loop system (9) are illustrated in Figure 6. From Figure 6, it is seen that when $E\left\{x^{T}\left(0\right)R_{i}x\left(0\right)\right\}=0<\rho_{1}=0.1$, $E\left\{x^{T}\left(k\right)R_{i}x\left(k\right)\right\}<\rho_{2}=2.6437$, which means that the CLS (9) is SFT $H_{\infty }$-bounded, that is to say, MJS (1) is SFT $H_{\infty }$ state feedback stabilization. Therefore, it is proven that the state feedback controller (8) designed in this paper is effective.

Next, the second example focuses on the effectiveness of the observer-based state feedback controller (32) designed in Theorem 4 for MJS (1) with partly unknown transition probabilities.

**Example** **2.** *The parameters of MJS (1) with three modes and partly unknown TPs are given as follows:*

*Mode 1 (i=1):*

*$A_{11}=\left[\begin{array}{cc} 0.1 & 0\\ 0 & -0.1 \end{array}\right],$ $A_{d11}=\left[\begin{array}{cc} 0.1 & 0\\ 0 & 0.1 \end{array}\right],$ $B_{11}=\left[\begin{array}{c} 1\\ -0.1 \end{array}\right],$ $C_{11}=\left[\begin{array}{c} 0\\ 0.1 \end{array}\right],$ $A_{21}=\left[\begin{array}{cc} -0.1 & 0\\ 0 & -0.2 \end{array}\right],$ $A_{d21}=$ $\left[\begin{array}{cc} 0.1 & 0\\ 0 & 0.1 \end{array}\right],$ $B_{21}=$ $\left[\begin{array}{c} -0.1\\ 0.1 \end{array}\right],$ $C_{21}=\left[\begin{array}{c} 0.1\\ 0.1 \end{array}\right],$ $D_{11}=\left[\begin{array}{cc} 0.5 & 0.1 \end{array}\right],$ $D_{d11}=\left[\begin{array}{cc} 0 & 0.5 \end{array}\right],$ $D_{1}=\left[\begin{array}{cc} 1 & 1 \end{array}\right],$ $G_{1}=1,$ $G_{11}=0.9$, $G_{21}=-0.1.$*

*Mode 2 (i=2):*

*$A_{12}=$ $\left[\begin{array}{cc} 0.1 & 0.1\\ 0.1 & 0.2 \end{array}\right],$ $A_{d12}=$ $\left[\begin{array}{cc} 0.2 & 0\\ 0 & 0.2 \end{array}\right],$ $B_{12}=$ $\left[\begin{array}{c} 0.1\\ 0.1 \end{array}\right],$ $C_{12}=$ $\left[\begin{array}{c} 0.1\\ 0.1 \end{array}\right],$ $A_{22}=$ $\left[\begin{array}{cc} 0.1 & 0\\ 0 & 0.1 \end{array}\right],$ $A_{d22}=$ $\left[\begin{array}{cc} 0.1 & 0\\ 0.2 & -0.1 \end{array}\right],$ $B_{22}=$ $\left[\begin{array}{c} 0.1\\ 0.1 \end{array}\right],$ $C_{22}=$ $\left[\begin{array}{c} 0.1\\ 0.1 \end{array}\right],$ $D_{12}=\left[\begin{array}{cc} 1 & 0.1 \end{array}\right],$ $D_{d12}=\left[\begin{array}{cc} 0.3 & 0.1 \end{array}\right],$ $D_{2}=\left[\begin{array}{cc} 2 & 1 \end{array}\right],$ $G_{2}=1,$ $G_{12}=1$, $G_{22}=0.1.$*

*Mode 3 (i=3):*

*$A_{13}=\left[\begin{array}{cc} 0.1 & 0\\ -0.2 & 0.1 \end{array}\right],$ $A_{d13}=\left[\begin{array}{cc} -0.2 & 0.2\\ 0.1 & 0.2 \end{array}\right],$ $B_{13}=\left[\begin{array}{c} 0.1\\ -0.1 \end{array}\right],$ $C_{13}=\left[\begin{array}{c} 0.1\\ 0.1 \end{array}\right],$ $A_{23}=\left[\begin{array}{cc} -0.2 & 0\\ 0 & -0.2 \end{array}\right],$ $A_{d23}=$ $\left[\begin{array}{cc} 0 & 0.2\\ 0.1 & 0.1 \end{array}\right],$ $B_{23}=$ $\left[\begin{array}{c} 1\\ 0.1 \end{array}\right],$ $C_{23}=\left[\begin{array}{c} 0.1\\ 0.1 \end{array}\right],$ $D_{13}=\left[\begin{array}{cc} 0.1 & -0.2 \end{array}\right],$ $D_{d13}=\left[\begin{array}{cc} 0.2 & 0 \end{array}\right],$ $D_{3}=\left[\begin{array}{cc} 1 & 1 \end{array}\right],$ $G_{3}=2,$ $G_{13}=2$, $G_{23}=0.1.$*

**Figure 7:**
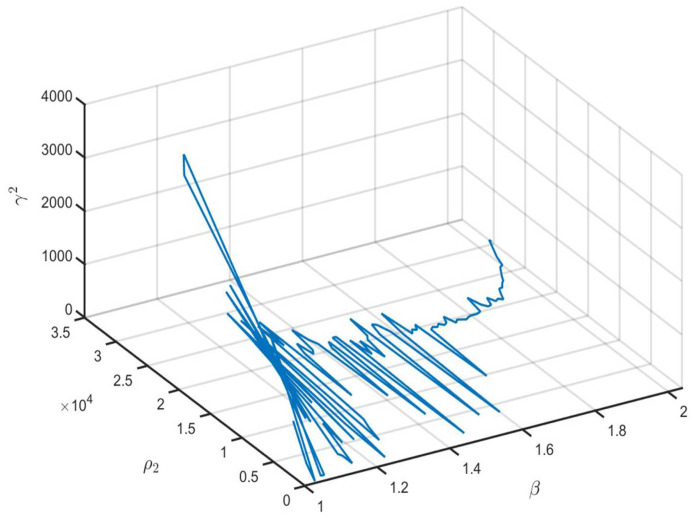
The optimal values of $\rho_{2}$ and $\gamma^{2}$ with different β values.

**Figure 8:**
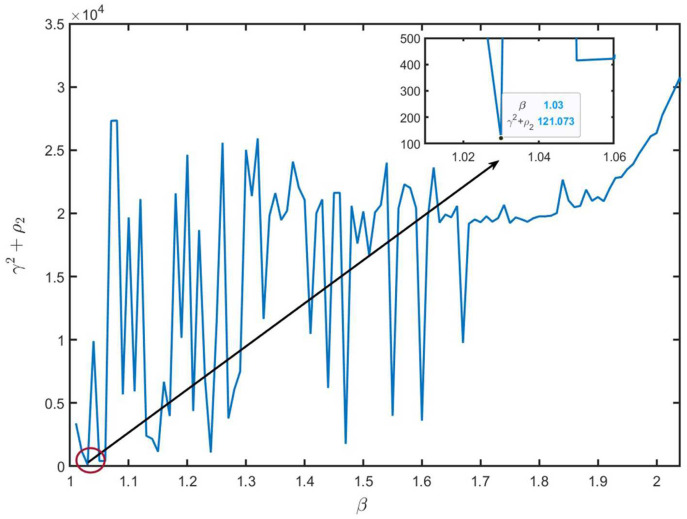
The values of $\gamma^{2}+\rho_{2}$ with different β values.

Then, letting $\varpi =10^{-10}$, ${\hat{R}}_{i}=I_{4\times 4}\;\left(i=1,2,3\right)$, the partly unknown TP matrix Π and the remaining parameters have identical values to those in Example 1. Similar to Example 1, we can obtain the feasible solution of (46) when 1.01≤β≤2.04. The relationships between β and $\gamma^{2}$ and $\rho_{2}$, and between β and $\gamma^{2}+\rho_{2}$ are shown in Figure 7 and Figure 8, respectively. From Figure 7 and Figure 8, we can see that the optimal values are $\rho_{2}=28.5424$ and $\gamma^{2}=92.5307$ with β=1.03.

Then, we compare the results of Theorem 4 with Theorem 2 in [44]. The optimal values of $\rho_{2}$ (i.e., τ in [44]) and γ obtained from the two works are shown in Table 2.

From Table 2, it appears that the optimal values of γ, $\rho_{2}$, and $\gamma^{2}+\rho_{2}$ obtained in this paper are smaller than those of [44], which indicates that the results of this paper are better. In addition, ref. [44] assumed that the transition probabilities were completely known, which means that the results of [44] are special cases of this paper.

In addition, by solving LMIs (37), (39), (40), (41), and (44), we have $\sigma_{2}=0.1779$, $\varrho_{1}=0.9901$, and $\varrho_{2}=5.2268$, and the gains of observer-based state feedback controller (32) are as follows:

$${\hat{K}}_{1}=\left[\begin{array}{cc} -0.2337 & -0.0433 \end{array}\right],\;{\hat{K}}_{2}=\left[\begin{array}{cc} -0.4995 & -0.0534 \end{array}\right],\;{\hat{K}}_{3}=\left[\begin{array}{cc} 0.0599 & 0.0414 \end{array}\right],$$

$$\;H_{1}=\left[\begin{array}{c} -0.0501\\ 0.0500 \end{array}\right],\;H_{2}=\left[\begin{array}{c} -0.0541\\ -0.0623 \end{array}\right],\;H_{3}=\left[\begin{array}{c} 0.0429\\ 0.0527 \end{array}\right].$$

**Figure 9:**
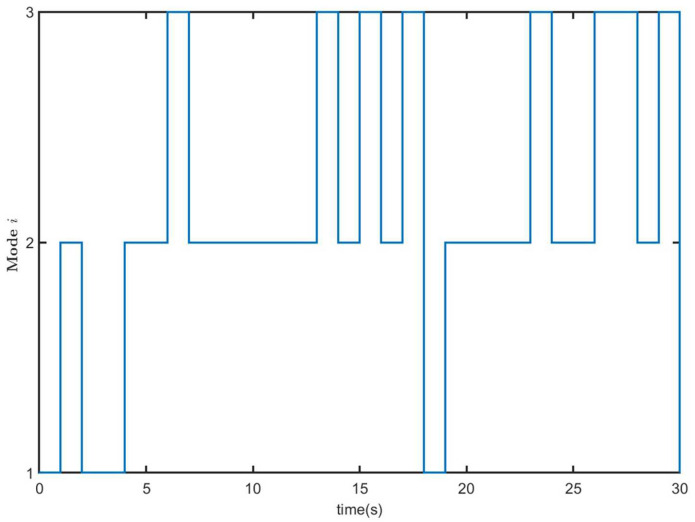
Markovian switching process of MJS (1) and CLS (33).

**Figure 10:**
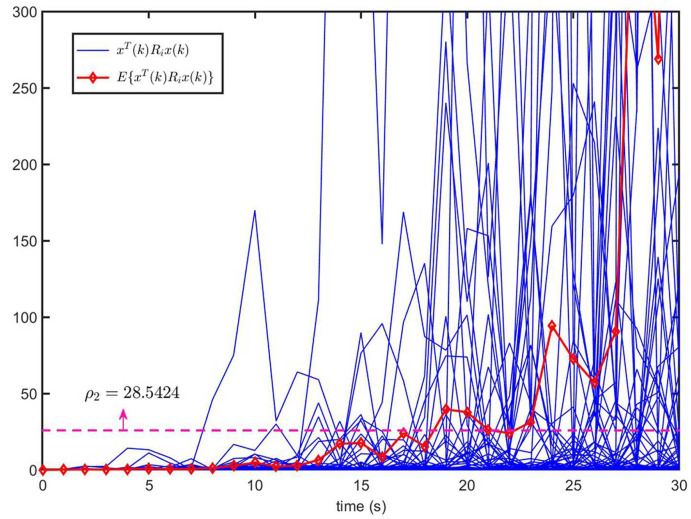
The trajectories of $x^{T}\left(k\right)R_{i}x\left(k\right)$ and $E\{x^{T}\left(k\right)R_{i}x\left(k\right)\}$ for open-loop system (1) (u(k)=0).

Next, we set the initial value $x\left(0\right)=\hat{x}\left(0\right)={\left[\begin{array}{cc} 0 & 0 \end{array}\right]}^{T}$ for systems (1) and (33), respectively. The external disturbance signal v(k) is the same as in Example 1. The Markovian switching process of MJS (1) and CLS (33) is shown in Figure 9. Figure 10 shows the trajectories of $x^{T}\left(k\right)R_{i}x\left(k\right)$ (50 curves) and $E\{x^{T}\left(k\right)R_{i}x\left(k\right)\}$ of open-loop system (1) (u(k)=0), which implies that open-loop system (1) is not finite time-bound.

**Figure 11:**
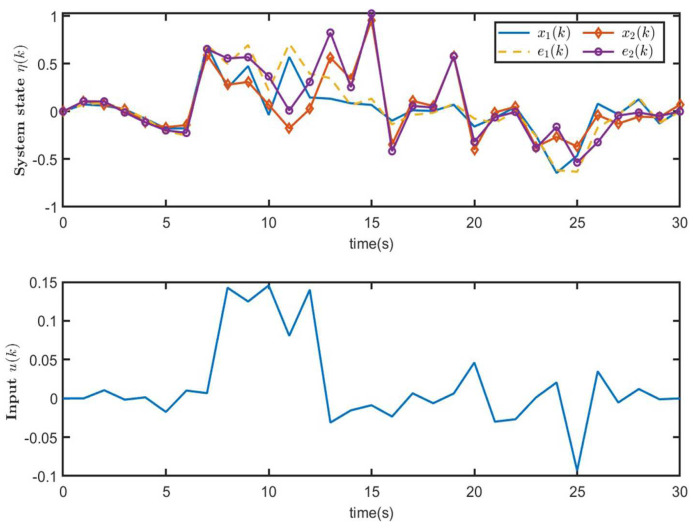
The trajectories of system state η(k) for CLS (33) and control input u(k) (32).

**Figure 12:**
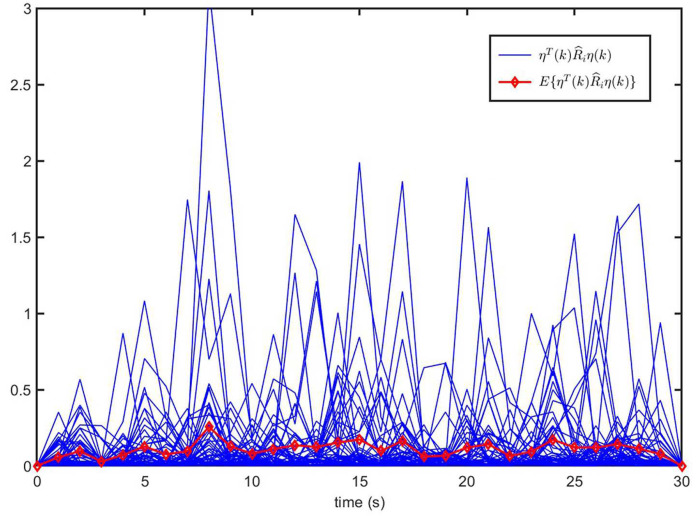
The trajectories of $\eta^{T}\left(k\right){\hat{R}}_{i}\eta \left(k\right)$ and $E\{\eta^{T}\left(k\right){\hat{R}}_{i}\eta \left(k\right)\}$ for CLS (33).

The trajectories of system state η(k) for CLS (33) and the curve of the control input u(k) of (32) are illustrated in Figure 11. Moreover, Figure 12 shows the trajectories of $\eta^{T}\left(k\right){\hat{R}}_{i}\eta \left(k\right)$ (50 curves) and $E\{\eta^{T}\left(k\right){\hat{R}}_{i}\eta \left(k\right)\}$ of closed-loop system (33). From Figure 12, it can be observed that CLS (33) is SFT $H_{\infty }$-bounded, i.e., MJS (1) is SFT $H_{\infty }$ observer-based state feedback stabilization. Furthermore, by comparing Figure 10 and Figure 12, it can be proven that observer-based state feedback controller (32) is effective.

## 6. Conclusions

Based on existing results, the design schemes of a stochastic finite-time $H_{\infty }$ state feedback controller and a stochastic finite-time $H_{\infty }$ observer-based state feedback controller for MJSs with a time delay and partly unknown TPs were studied in this paper. A state feedback controller and an observer-based state feedback controller were designed and some sufficient conditions for the CLSs to satisfy SFT $H_{\infty }$ boundedness were presented via LKF technology. Then, the controller gains were obtained by using the LMI method. Lastly, two examples were provided to verify the validity of the proposed design schemes. In the following work, the finite-time guaranteed cost control and event-triggered control of discrete-time MJSs will be studied on the basis of this paper.

## Figures and Tables

**Table 1:** The acronyms used in this article and their meanings.

Acronyms	Meaning of Acronyms
MJS	Markovian jump system
SFT	Stochastic finite-time
LKF	Lyapunov–Krasovskii functional
CLS	Closed-loop system
LMI	Linear matrix inequality
TP	Transition probability

**Table 2:** The optimal values of γ, $\rho_{2}$, and $\gamma^{2}+\rho_{2}$.

Method	Theorem 4 in This Paper	Theorem 2 in Reference [44]
γ	9.6193	105.9526
$\rho_{2}$	28.5424	150.4146
$\gamma^{2}+\rho_{2}$	121.0731	11,376.368

## Data Availability

The data presented in this study are available on request from the corresponding author.
